# Supplementary material for: CXCL12α/SDF‐1 from perisynaptic Schwann cells promotes regeneration of injured motor axon terminals
Source: EMBO Mol Med. 2017 May 30;9(8):1000–10. doi: 10.15252/emmm.201607257 (PMC5538331; doi:10.15252/emmm.201607257)
Supplement: Supplementary file 1 — Expanded View Figures PDF [file EMMM-9-1000-s001.pdf]

Expanded View Figures

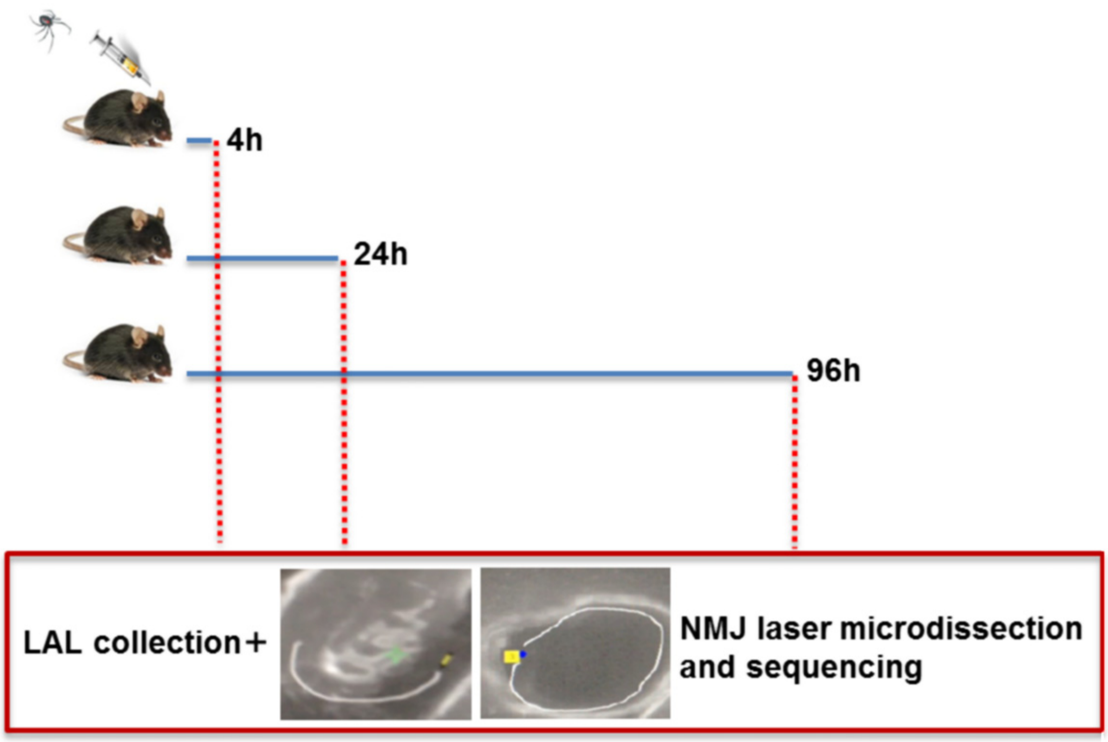

Figure EV1. Outline of NMJ transcriptome analysis.
Mice were injected with α-LTx in proximity to LAL and fixed at different time points during MAT degeneration and regeneration, defined on the basis of the kinetics reported in Fig 1A and B. After muscle collection, 50 NMJs/sample were laser-microdissected, pooled, and processed for NGS (next-generation sequencing).

© 2017 The Authors

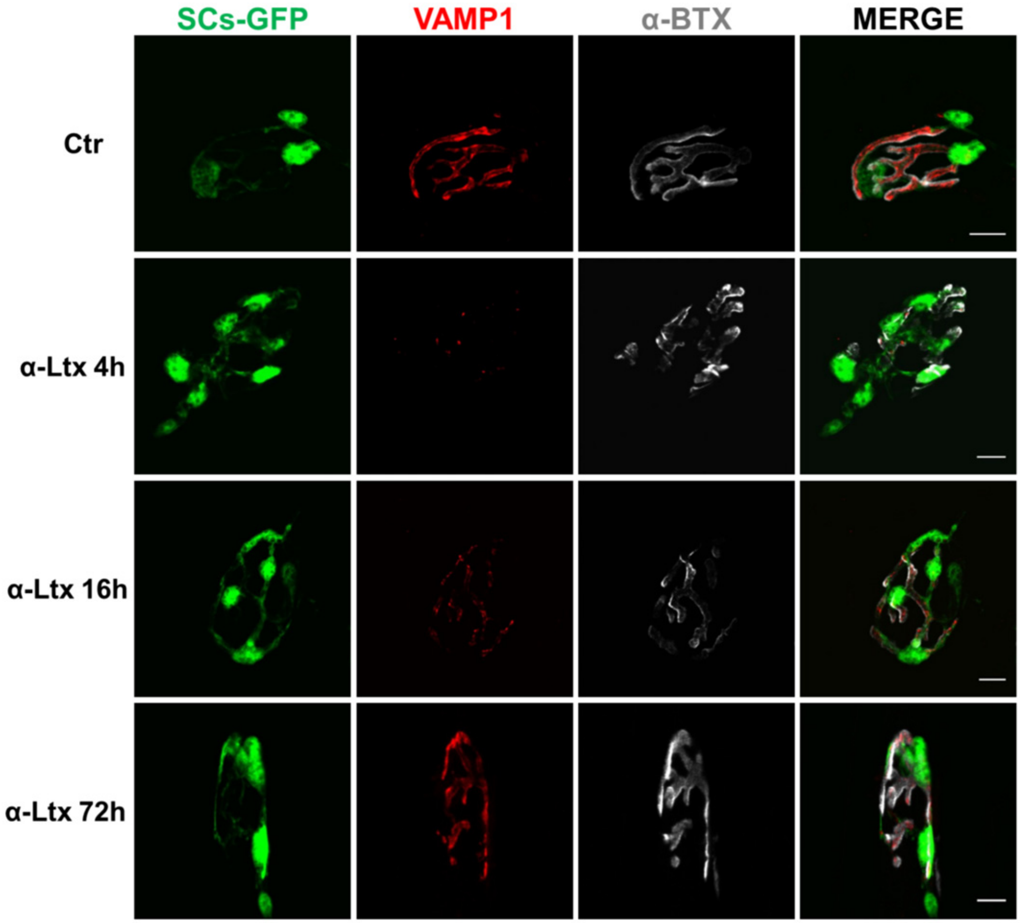

Figure EV2. Kinetics of nerve terminal degeneration and regeneration in soleus muscle.
The time course of MAT degeneration and regeneration induced by α-LTx at soleus NMJs was determined in mice with GFP-expressing SCs (green), using the presynaptic marker VAMP1 (red). The post-synaptic differentiations are identified by α-bungarotoxin (α-BTx) staining (white). Muscles were fixed 0, 4, 16, and 72 h post-injection. Scale bars: 10 μm.

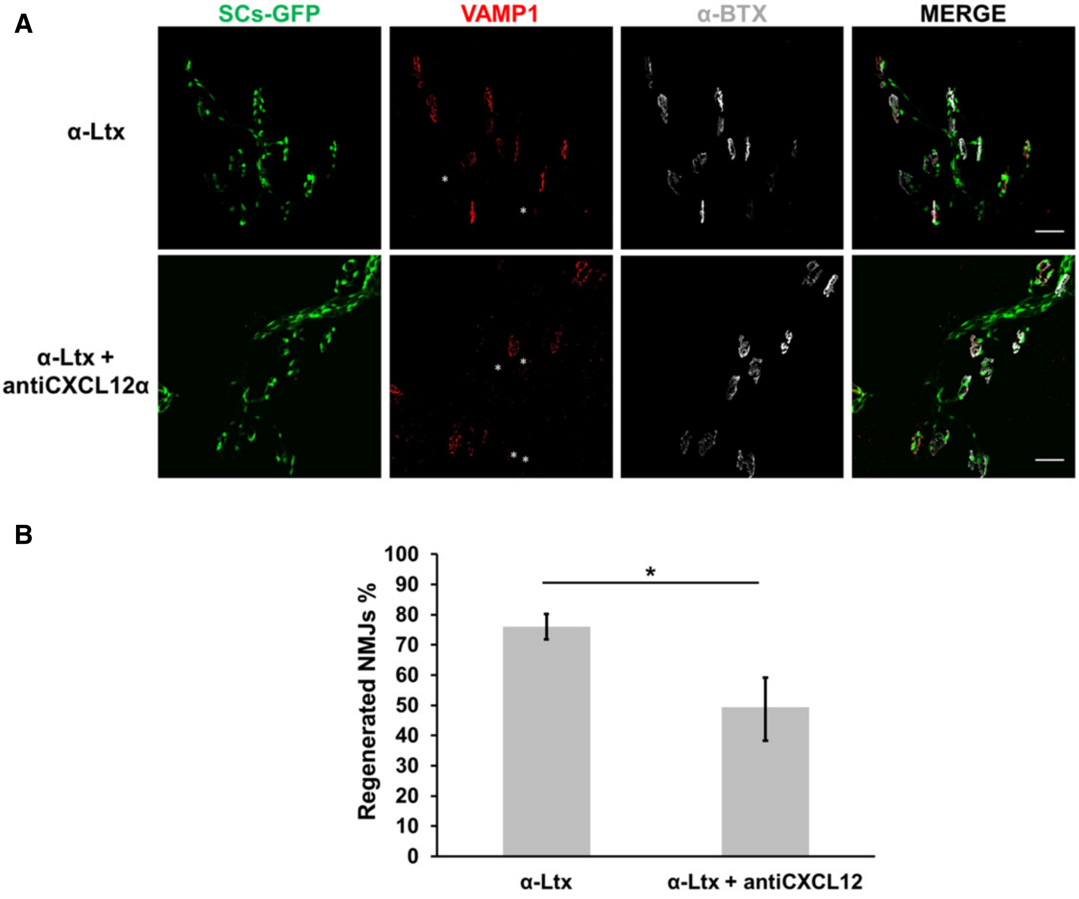

Figure EV3. CXCL12α neutralization slows down nerve terminal regeneration.

A, B Anatomical recovery of soleus NMJs 72 h after α-LTx injection in the mice hind limb, with/without a previous intraperitoneal administration of a CXCL12α-neutralizing antibody, as assessed by immunostaining the presynaptic marker VAMP1 (red). GFP-expressing SCs are in green. The post-synaptic differentiations are identified by α-bungarotoxin (α-BTx) staining (white). Asterisks identify those NMJs that are still degenerated (positive for α-BTx but negative for VAMP1). Scale bars: 10 μm. Quantitation is shown in (B). Data are presented as mean ± SD. *P = 0.0123 by Student's t-test, unpaired, two-sided.

 © 2017 The Authors

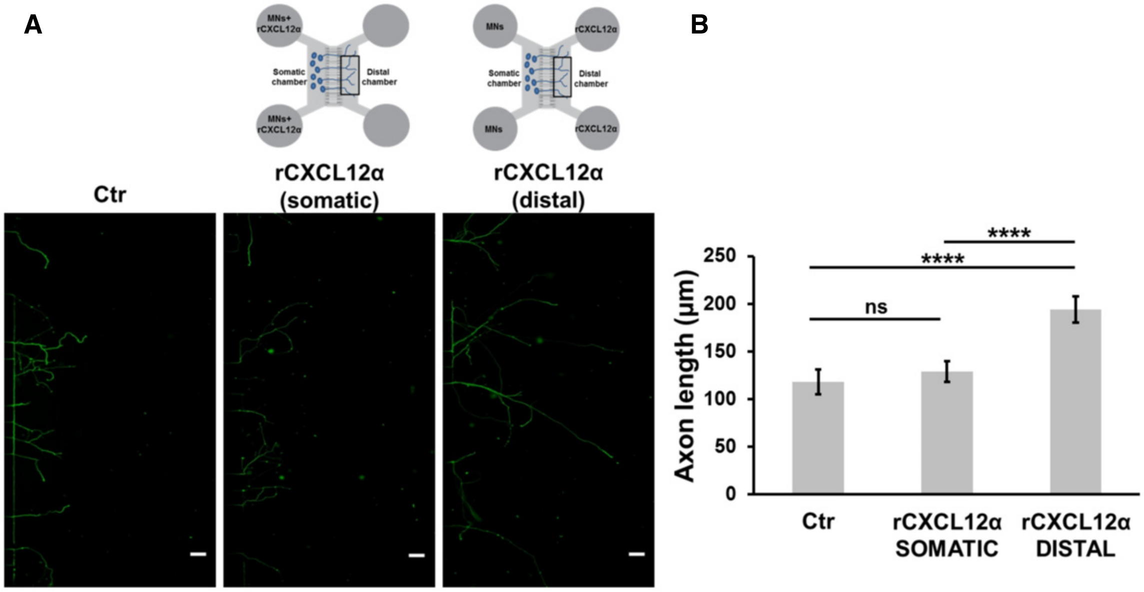

Figure EV4. Recombinant CXCL12α fails to promote axonal elongation when added to neuronal soma compartment.

A, B *r*CXCL12 added to the somatic compartment of microfluidic devices fails to promote axonal elongation of SCMNs. The figure shows the distal chamber after 5 days of culture. Scale bars: 50 μm. Panel (B) shows the quantification of axon growth from three experiments. Seventy neurons measured per experiment. Data are presented as mean ± SD. ****$P < 0.0001$ by ANOVA followed by *post hoc* Tukey test. ns = not significant.

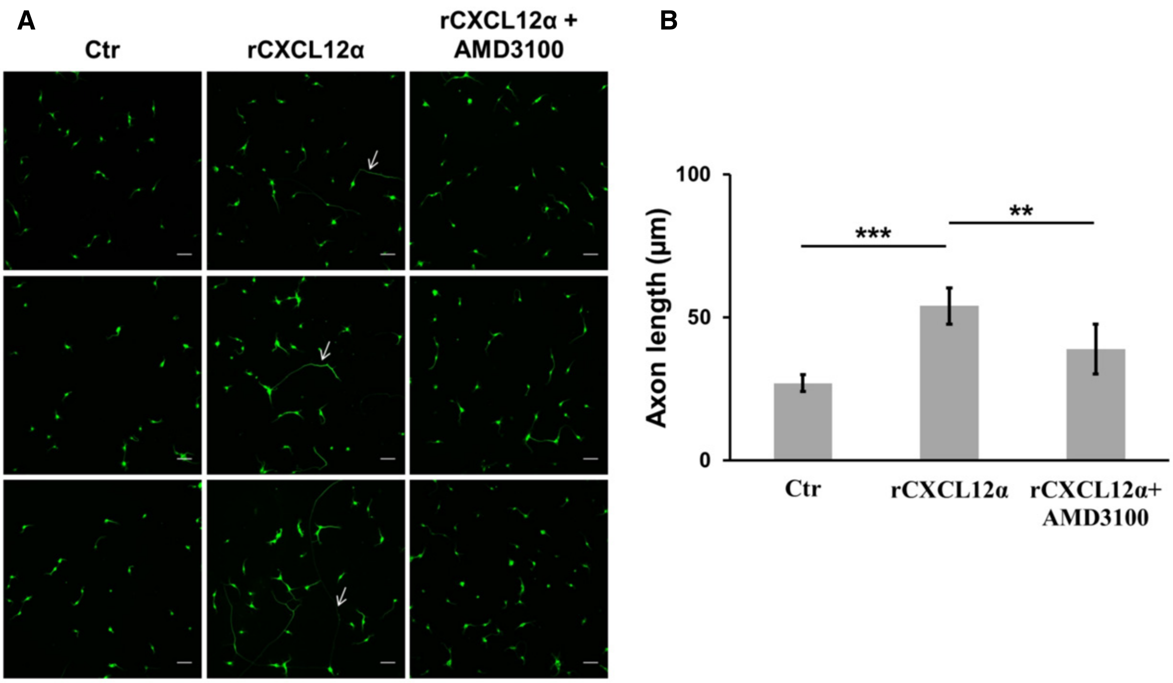

Figure EV5. CXCL12α promotes axon growth via CXCR4.

A, B *r*CXCL12α was added to SCMNs plated in culture dishes and axon elongation was monitored for 24 h. The axon growth-stimulating ability of the chemokine was reduced in the presence of AMD3100. Scale bars: 50 μm. Quantification is shown in (B). Each bar represents mean ± SD from five different experiments, 70 neurons measured per experiment. ***$P = 0.0003$, **$P = 0.0061$ by ANOVA followed by *post hoc* Tukey test. Arrows indicate single axons to point out the elongating effect exerted by the chemokine.

© 2017 The Authors
